# Ligand Decomposition Differences during Thermal Sintering of Oleylamine-Capped Gold Nanoparticles in Ambient and Inert Environments: Implications for Conductive Inks

**DOI:** 10.1021/acsanm.3c04803

**Published:** 2023-12-13

**Authors:** Kai Chang, Chinmoy Podder, Heng Pan

**Affiliations:** J. Mike Walker’66 Department of Mechanical Engineering, Texas A&M University, College Station, Texas 77843, United States

**Keywords:** gold nanoparticles, conductive inks, ligand decomposition, printable electronics, sintering, oleylamine

## Abstract

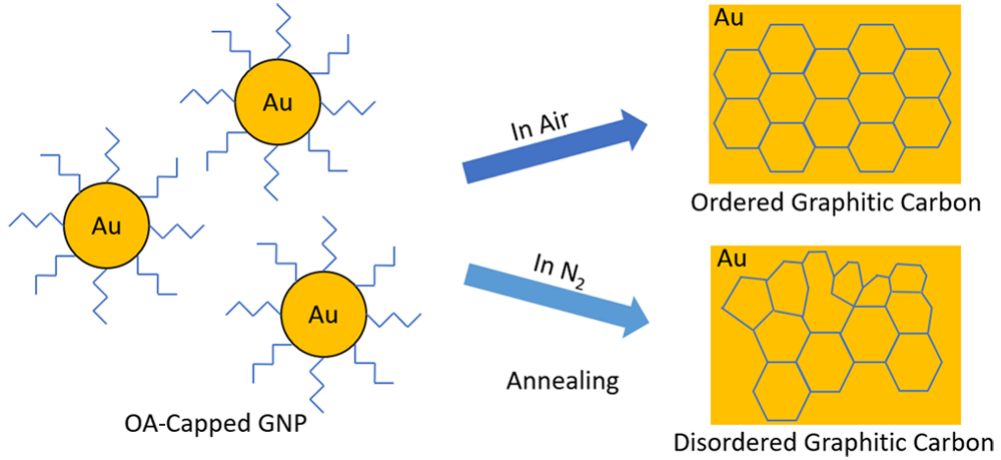

Gold nanoparticles (GNPs) are essential in creating conductive inks vital for advancing printable electronics, sensing technologies, catalysis, and plasmonics. A crucial step in fabricating useful GNP-based devices is understanding the thermal sintering process and particularly the decomposition pathways of ligands in different environments. This study addresses a gap in the existing research by examining the sintering of oleylamine (OA)-capped GNPs in both ambient (air) and inert (N_2_) environments. Through a series of analyses including TGA/MS, Raman spectroscopy, and XPS, distinctive OA decomposition behaviors were identified in air and nitrogen environments. The research delineates two OA decomposition pathways resulting in different porosity, microstructure, and electrical conductivity of GNP films sintered in air and nitrogen environments. The study offers some insights that can steer the sintering and utilization of the GNP sintering process and promises to aid the future development of nanoparticle-based printable electronics.

## Introduction

Gold nanoparticles (GNP) have garnered significant attention in contemporary nanotechnology, underpinned by their unique properties such as easy solution processability,^1^ robust plasmonic absorption, and efficient light scattering.^2,3^ This attention is substantiated by their varied applications in realms like photothermal therapy,^4,5^ thermoelectrics,^6,7^ photovoltaics,^8,9^ and photocatalysis.^10−13^ Traditionally, the gold nanoparticles are synthesized as a colloidal suspension, commonly referred to as “ink”, which encompasses ligands to prevent agglomeration and maintain the desired nanoparticle size and shape. Numerous ligands have been used in gold nanoparticle synthesis such as short-chain thiols,^14,15^ amines,^14,16^ and phosphine,^17^ and different ligand types will help to fulfill different applications.

The gold nanoparticles have great potential in many fields that require conductive gold patterns such as printable electronics and additive manufacturing.^18−21^ In these applications, the capping ligands on the nanoparticle surface need to be removed to achieve properties of bulk gold.^22−24^ Many printing techniques^25−28^ have been developed over the years, most of which rely on post-treatment steps to remove the ligands and sinter the printed patterns. The techniques centered around the removal of organic ligands from the surface of these nanoparticles have been reported extensively, outlining a series of approaches including thermal treatments in various environments,^29−36^ treatments augmented by techniques such as plasma,^37,38^ UV-ozone,^39^ and chemical assistance,^40,41^ as well as solvent extraction techniques.^42^ Despite considerable advancements, work addressing the formation of carbon residues following ligand removal has been lacking. The residue will heavily influence the printed pattern properties such as morphology, microstructures, and conductivity; thereby, knowing the residues and their influences is critically important in improving the functions of printed electronics.

Among the different ligands, oleylamine (OA) is widely used to cap gold nanoparticles for its versatile functions such as capping ligand, reducing agent, surfactant, and stabilizer.^43−46^ It is suitable for printable electronics and additive manufacturing because of its low binding energy with surface gold to achieve low-cost removal, especially by thermal decomposition. Although OA has been extensively used in nanoparticle synthesis, there are not enough reports discussing its exact thermal decomposition route upon thermal sintering.

In this paper, OA-capped gold nanoparticle films have been investigated to understand the thermal decomposition of ligands and their residual organic constituents during sintering in air and N_2_. TGA coupled mass spectroscopy revealed that different thermal events happen between sintering in N_2_ and air and various species generated at sintering temperatures. Distinctly different transformations of the ligand, which leads to the formation of short-range ordered nanocrystalline graphitic carbon in air and the formation of disordered nanocrystalline graphitic carbon in a N_2_ environment, were revealed via Raman spectroscopy. Furthermore, optical and electrical properties of the sintered film in different environments were characterized. This understanding is expected to provide insight into controlling the ligand decomposition in partially and fully sintered inorganic nanoparticle films for various applications.

## Experimental Methods

### Nanoparticle Ink

Oleylamine-capped gold nanoparticle ink (3–5 nm particle size in Xylene, UTDAu25X) was obtained from UTdots. The synthesis recipe of this ink is mentioned in the literature.^47^ Technical grade (70%) oleylamine was obtained from Sigma-Aldrich.

### Thin Film Deposition

A Laurell WS 650-23B spin coater was used to perform the spinning process for thin film deposition. First, a regular glass slide was cut into smaller pieces and cleaned with different solvents including acetone, ethanol, isopropanol, and deionized water. Highly concentrated (25 wt %) commercial OA-capped gold nanoparticle ink was then spin-coated on the cleaned glass substrates. Varying speeds of 1000, 2000, 4000, and 6000 rpm and a fixed time of 45s were set as the spinning program to achieve deposited films with varying thickness.

### Electron Microscopy (SEM/TEM)

SEM images were captured by using a scanning electron microscope (Tescan LYRA-3 Model GMH focused ion beam microscope). TEM images were captured using a Tecnai F20 TEM machine.

### Fourier Transform Infrared Spectroscopy (FTIR)

Nicolet 4700 FT-IR was used to measure the spectra from OA and OA-capped ink to confirm the presence of the ligand in the ink.

### Raman Spectroscopy

Raman measurements were carried out using Horiba Jobin-Yvon LabRam HR with a 633 nm HeNe laser as the excitation source with its 100% power equaling 11.16 mW (average reading from powermeter). For attaining the Raman spectra for this study, this laser power was varied by using different filters ranging from 0.5% to 50% of the total power. A 50X objective lens was used on the desired sample area to attain the spectra.

### Thermogravimetric Analysis and Mass Spectrometry (TGA/MS)

TGA data were obtained using a TGA 5500 TA instrument. Here, a 20 °C/min heating rate was used up to 800 °C. A TGA chamber nitrogen environment was attained by a 100 mL/min gas flow rate for purging for 10 min and then 25 mL/min for the test. For TGA data in air, the same procedure was used with air as the sampling gas. MS data were obtained with a Discovery mass spectrometer coupled with TGA to scan species from the TGA ranging from 10 to 100 *m*/*z* with a cycling time of 5.2 s.

### X-ray Photoelectron Spectroscopy (XPS)

An XPS measurement was carried out with SPECS EnviroESCA to detect surface elements and corresponding binding properties. Here, 25 wt % gold ink was dripped on glass substrates and heated in nitrogen and air, respectively, for 10 min. The samples were placed on the stage of XPS, and an XPS measurement was carried out with a 0.05 eV increment. The acquired data were processed with CasaXPS software.

### Optical Transmission Spectroscopy

Optical transmission spectroscopy was performed using an Ocean Optics spectrometer (USB 2000+) to obtain transmittance spectra of the deposited thin films. Here, 25 wt % gold ink was spin-coated on to glass substrates and then placed on a stage in between a white light source and the spectrometer lens to obtain spectra of different temperature-annealed gold films.

## Results and Discussion

**Figure 1:**
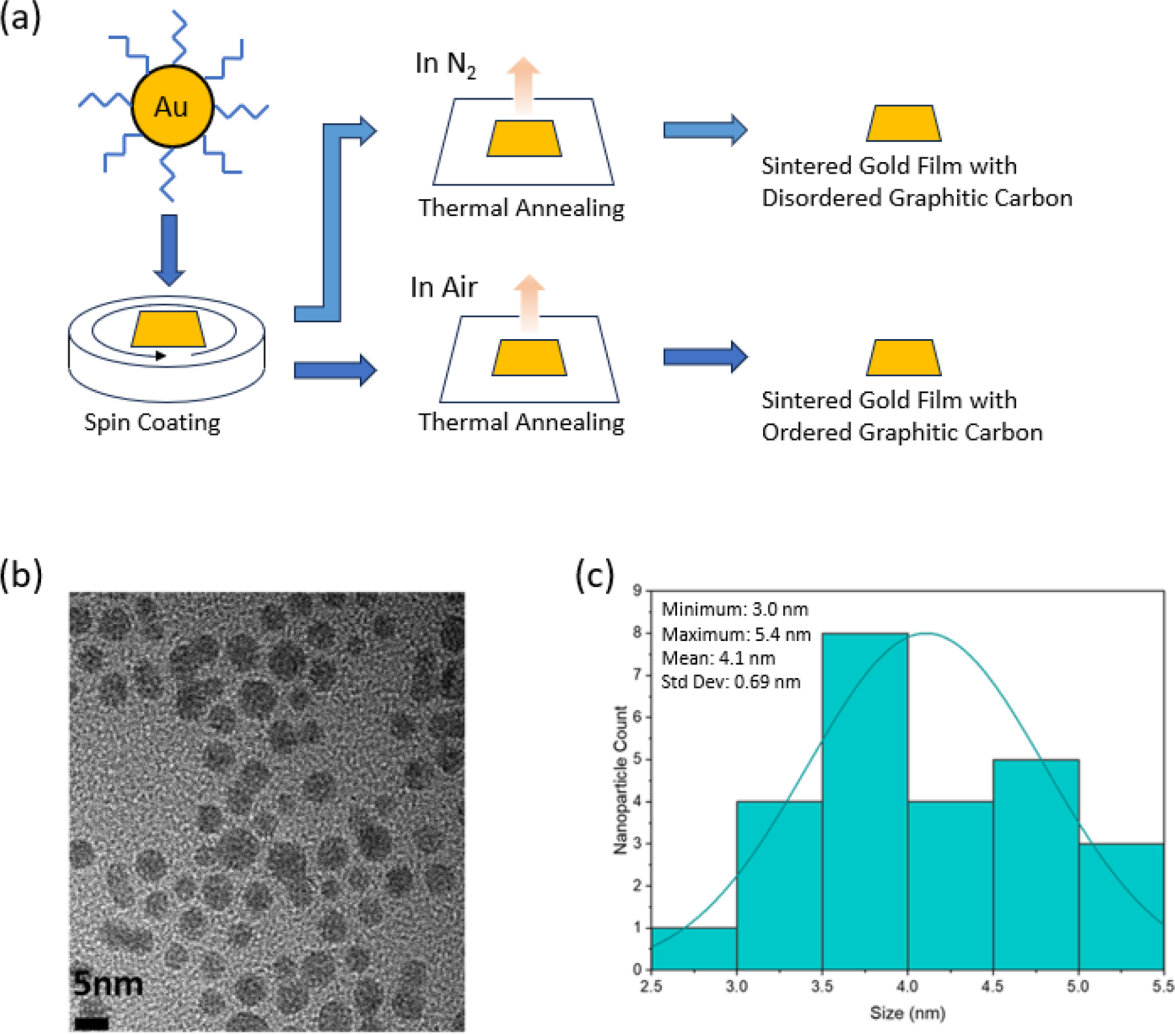
(a) Thermal annealing of gold film in air and N_2_ environments. (b) TEM image (scale bar 5 nm) of OA-capped gold nanoparticles from the as-deposited ink and (c) particle size histogram showing average nanoparticle size of ∼4 nm.

This work mainly focuses on the decomposition path of the ligand on the gold nanoparticles when performing the thermal annealing treatment of the GNP film. The typical process of the thermal annealing is shown in Figure 1(a). First, the GNP ink was spin-coated on a glass substrate, and then, the samples were heated on hot plates in air and N_2_ environments. After thermal annealing, conductive gold films were obtained with different residues resulted from ligand decomposition. The ligand molecule (OA) is an 18-carbon long chain with a C=C exactly at the middle and an amine functional group at the head. The ligand typically covers the NP from all sides to retain the actual size/shape and its colloidal stability. The OA-capped GNP used in this study was characterized to verify the particle size distribution. Figure 1(b) demonstrates a TEM image of the spherical GNP. The average size of the NP was measured to be ∼4 nm according to the particle size histogram in Figure 1(c). It is also observed that the NPs are separated by a distance from each other. This is due to the steric repulsion of the encapsulating ligands on the surface of the NPs.

**Figure 2:**
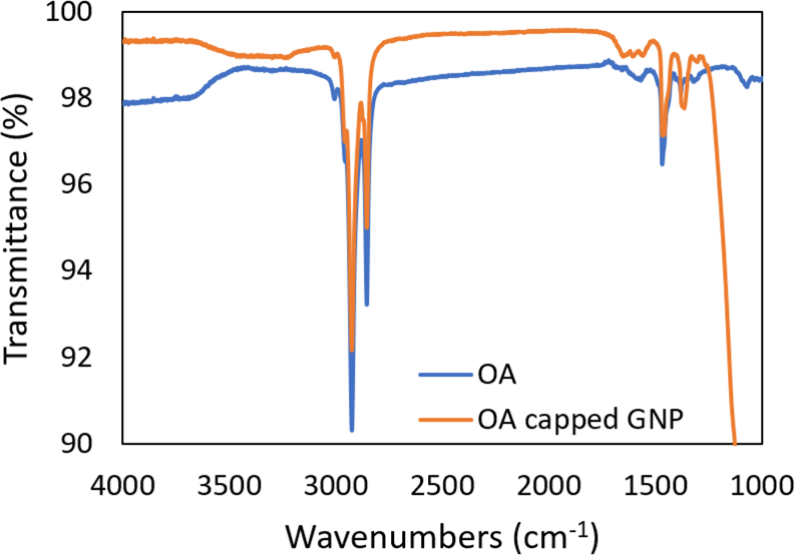
Fourier transform infrared spectroscopy (FTIR) of OA and OA-capped gold ink.

To verify the presence of OA as the capping ligand in the ink, we used FTIR spectroscopy to analyze OA and OA-capped GNP ink spectra (Figure 2). Strong peaks were observed at 2940, 2922, and 2851 cm^–1^ in the FTIR spectra of the OA-capped GNP sample attributed to the CH_2_ symmetric and asymmetric stretching. The peaks at 1560 and 1655 cm^–1^ corresponded to the NH_2_ bending vibration and C=C bending vibration, respectively. These results confirmed the presence of oleylamine in the GNP film.

**Figure 3:**
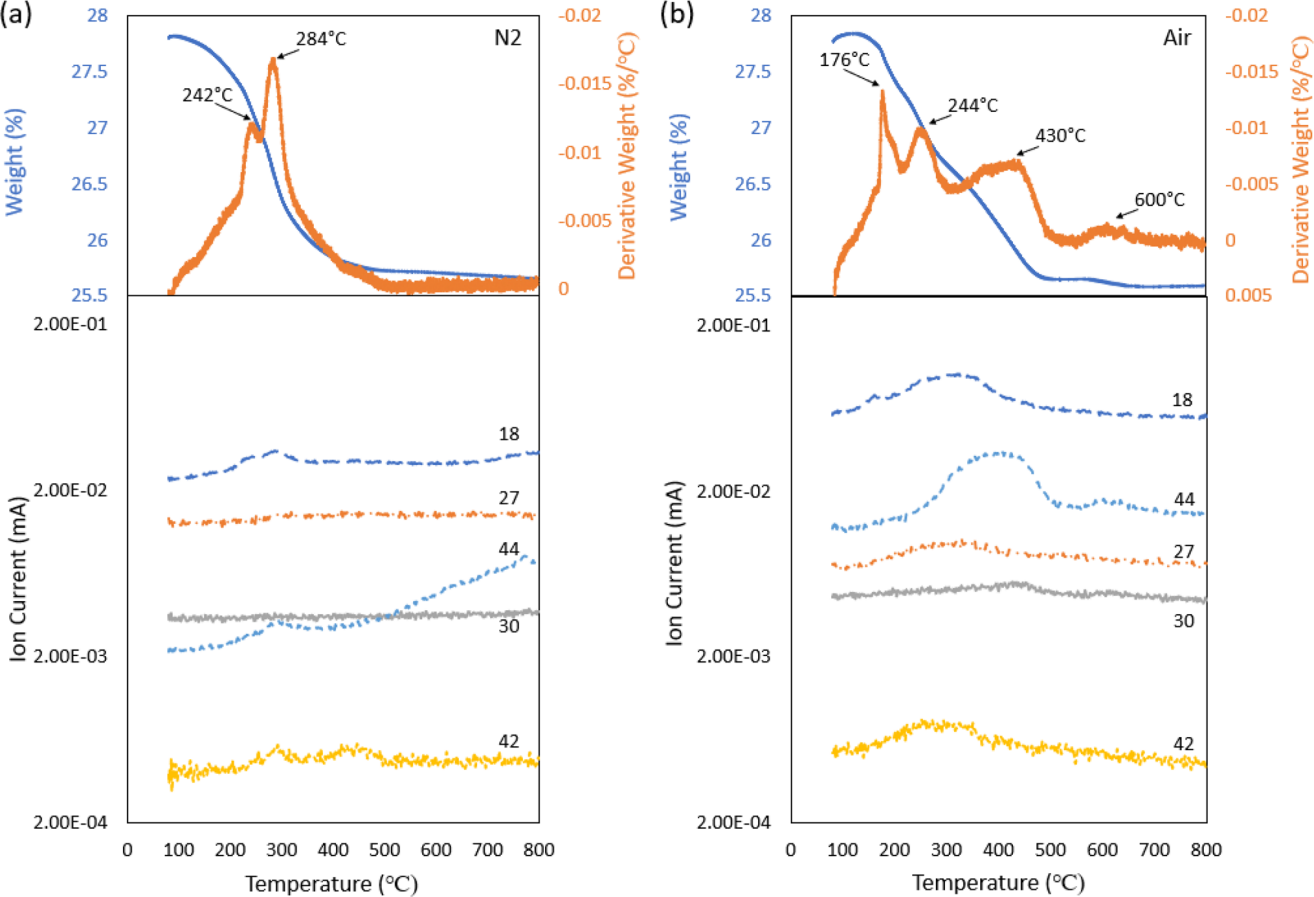
(a) TGA (top) and mass spectrum (bottom) of OA-capped gold ink in N_2_. (b) TGA (top) and mass spectrum (bottom) of OA-capped gold ink in air.

To provide the comparison of mass loss and corresponding species generation from the OA and OA-capped GNP ink during thermal sintering, TGA/MS was used to analyze the data for both the air and N_2_ environments. All the samples were purged with N_2_ and air, respectively, for 10 min before sample heating and collecting TGA/MS data. The OA samples were ramp heated to 800 °C with a 20 °C/min heating rate, and GNP ink samples were first heated to 80 °C and maintained for 15 min to evaporate the solvent (xylene) and then heated to 800 °C with a 20 °C/min rate. Figure 3(a) and (b) depict the mass change and corresponding first derivative of mass change for the GNP ink sample heated from 80 to 800 °C in N_2_ and air, respectively. Note in both plots the weight percentage starts at around 28% which is close to the nominal weight percentage of gold nanoparticles in the ink considering most of solvent (xylene with weight percentage ∼82%) is evaporated at the end of the initial temperature ramping to 80 °C. Thus, the mass drops (from ∼28% to ∼25.5%) as seen in Figure 3 are owed to the ligand decomposition.

Besides the mass change, its first order derivative of mass change with respect to the temperature is also presented, which is known as derivative thermogravimetry (DTG). DTG shows the mass loss rate at a given temperature, and the peaks indicate thermal events such as material evaporation, pyrolysis, decomposition, and/or chemical reaction with volatile species generation.^48^ When annealed in air, the ink sample shows mass loss mainly at ∼240 and 284 °C with two distinct peaks, and when heated in air, the ink shows four peaks at ∼176, 244, 430, and 600 °C. The difference in peak number and position indicates different thermal events occurring during annealing in the two different environments. The two higher peaks in the N_2_ environment suggests the mass loss in N_2_ is higher than in air at relatively lower temperatures (<300 °C). This is in agreement with a study^49^ on similar GNP (supported on TiO_2_) that reported that TGA mass loss in N_2_ can be higher than air which matches with our observation. Significant mass loss continues to be observed after ∼300 °C for the sample annealed in air as indicated by the third and fourth peaks.

To better understand the thermal events that occurred during the annealing, mass spectrometry (MS) was coupled with TGA to analyze the generated species from the GNP ink sample upon heating. Particles with mass-to-charge ratio (*m*/*z*) in the range from 10 to 150 were recorded, and species with detectable changes (*m*/*z* 18, 27, 30, 42, 44) are presented in Figure 3. In the N_2_ environment, H_2_O^+^ (*m*/*z* 18), C_3_H_6_^+^ (*m*/*z* 42), and CO_2_^+^ (*m*/*z* 44) show noticeable peaks at ∼200–300 °C that partially coincide with the DTG peaks, while a slight change of C_2_H_3_^+^ (*m*/*z* 27) was detected, and amine CH_2_NH_2_^+^ (*m*/*z* 30) remained nearly flat during the entire annealing process. In the air environment, on the other hand, the signals from *m*/*z* 18 and 44 species are significantly higher as compared with N_2_ annealing, which implies significantly more H_2_O^+^ and CO_2_^+^ have been generated and released upon annealing in air. Similar to annealing in N_2_, C_3_H_6_^+^ (*m*/*z* 42) shows a peak that partially overlaps with DTG peaks. It is worth noting that the change of C_2_H_3_^+^ (*m*/*z* 27) and, in particular, the amine CH_2_NH_2_^+^ (*m*/*z* 30) and their correlation with DTG peaks were found to be more obvious in air as compared to those in N_2_.

The common species that can be detected in both environments include H_2_O^+^ (*m*/*z* 18), C_2_H_3_^+^ (*m*/*z* 27), C_3_H_6_^+^ (*m*/*z* 42), and CO_2_^+^ (*m*/*z* 44). The rise of these species partially coincides with the DTG peaks, suggesting that these species are generated upon thermal heating and ligand decomposition. One major difference is that the amount detected is higher in air. Both environments generated species H_2_O^+^, while in the N_2_ environment without oxygen, it is impossible to generate H_2_O^+^. Possible explanations are that oxygen or water could be introduced during gold nanoparticle synthesis or by the technic grade oleylamine that contains an unknown solvent. Another noticeable difference between the two environments is that correlation of amine CH_2_NH_2_^+^ (*m*/*z* 30) with DTG peaks is much more obvious in air annealing. Amine CH_2_NH_2_^+^ (*m*/*z* 30) is commonly used in the literature as a signature for amine molecules.^50−52^ This suggests that the annealing environment affects the amine decomposition and removal process. The detection of CH_2_NH_2_^+^ (*m*/*z* 30), C_3_H_6_^+^ (*m*/*z* 42), and CO_2_^+^ (*m*/*z* 44) continue to be observed at higher temperatures (300–600 °C) when annealing in air. In particular, the coincidence of strong CO_2_^+^ and amine CH_2_NH_2_^+^ peaks with the third and fourth DTG peaks suggests they were generated due to the OA decomposition at ∼430 and ∼600 °C.

In summary, the distinct differences between air annealing and N_2_ annealing lie the following: (1) In a N_2_ environment, the mass loss mainly occurs below 400 °C while mass loss in air continues to be observed up to 600 °C. (2) Higher levels of H_2_O^+^ (*m*/*z* 18), C_2_H_3_^+^ (*m*/*z* 27), C_3_H_6_^+^ (*m*/*z* 42), and CO_2_^+^ (*m*/*z* 44) were observed in air annealing as compared with N_2_ annealing, and change of amine CH_2_NH_2_^+^ (*m*/*z* 30) is only detectable in air annealing, which indicates the decomposition path and products are different in these two environments.

**Figure 4:**
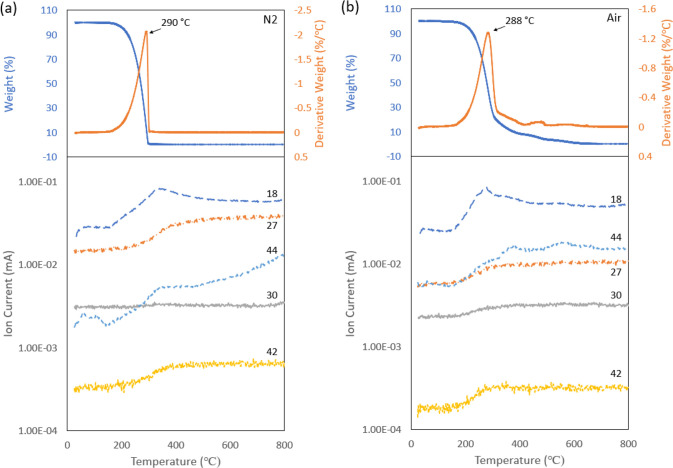
(a) TGA (top) and mass spectrum (bottom) of OA in N_2_. (b) TGA (top) and mass spectrum (bottom) of OA in air.

In addition, the TGA coupled mass spectrum of heating OA in the two environments was also obtained as reference and presented Figure 4. Both DTG curves show only one main peak, and it was found that OA mass loss started at ∼190 °C and completed (weight% reduced to 0) at ∼290 °C in N_2_ (the DTG peak falls drastically to almost 0 at ∼290 °C). In contrast, in air, TGA shows that at ∼288 °C the weight reduced to ∼20%, and its DTG peak remains above 0 until ∼600 °C, which indicates the higher temperature decomposition of oleylamine.

Interestingly, the starting temperature of OA mass loss is reduced by ∼50 °C in OA-capped GNP ink compared to that in OA when heated in N_2_. The temperature shift is larger when heated in air (∼110 °C). It has been reported in the literature that catalysis of metal (Pt) nanoparticles can induce thermal degradation of the adsorbed molecules at a lower temperature than the temperature needed for degrading a pure (adsorbed) molecule.^53,54^ Similarly in our study, the observed reduced mass loss start temperature of OA in GNP can be attributed to the catalysis of Au.

The mass spectra were also collected for OA heating in two environments, as presented in Figure 4. It can be seen that the MS spectra and detected species of OA (Figure 4) appear to be similar to the GNP ink sample (Figure 3). Common species can be detected in both N_2_ and air environments including H_2_O^+^ (*m*/*z* 18), C_2_H_3_^+^ (*m*/*z* 27), C_3_H_6_^+^ (*m*/*z* 42), and CO_2_^+^ (*m*/*z* 44). The rise of these species generally coincides with the first DTG peak (at 290 °C in N_2_ and 288 °C in air). This coincidence suggests these species are generated upon thermal heating of OA. It is also interesting to note that the rise of amine CH_2_NH_2_^+^ (*m*/*z* 30) is noticeable only in air annealing, which aligns with the result of GNP ink annealing.

**Figure 5:**
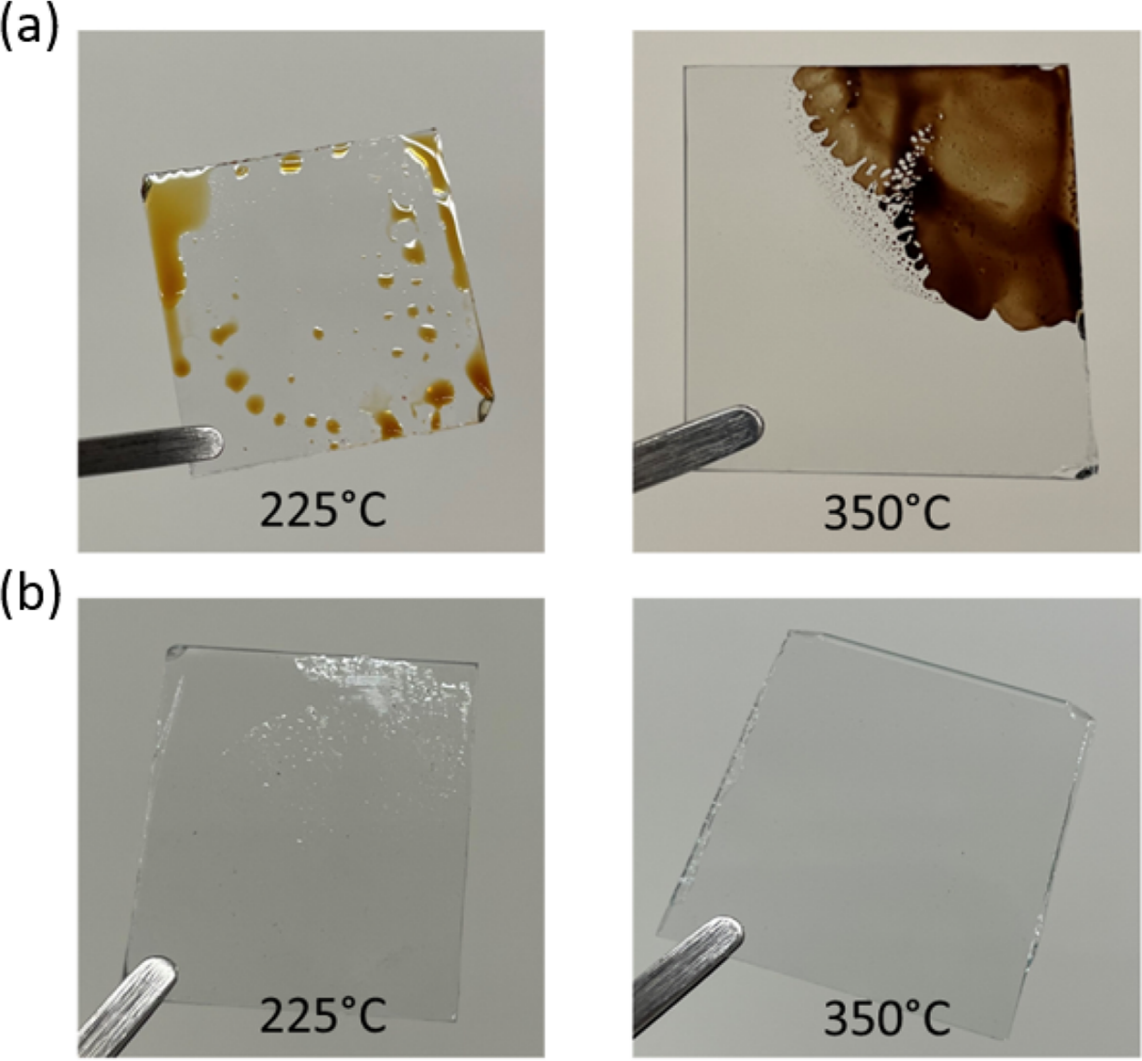
Oleylamine (OA) heated in (a) air and in (b) N_2_ environment for 10 min showing difference in color.

Besides TGA/MS, the OA decomposition was visually examined during hot plate heating at two different temperatures in the air and N_2_ environments. Figure 5(a) depicts the OA color change under air heating at 225 °C (light brown) and 350 °C (dark brown). This color change for air heating of OA is in correspondence with the literature.^55^ In contrast, OA heating in N_2_ (Figure 5(b)) shows almost no color change at both temperature heating. At 225 °C, there is partial mass loss in N_2_ (perceived from TGA) which can be confirmed by residual colorless OA presence on the glass slides, whereas at 350 °C, OA is completely removed, and no visible residue is observed on the glass slide. This difference aids in the fact that OA thermal decomposition is distinctly different in air and N_2_.

**Figure 6:**
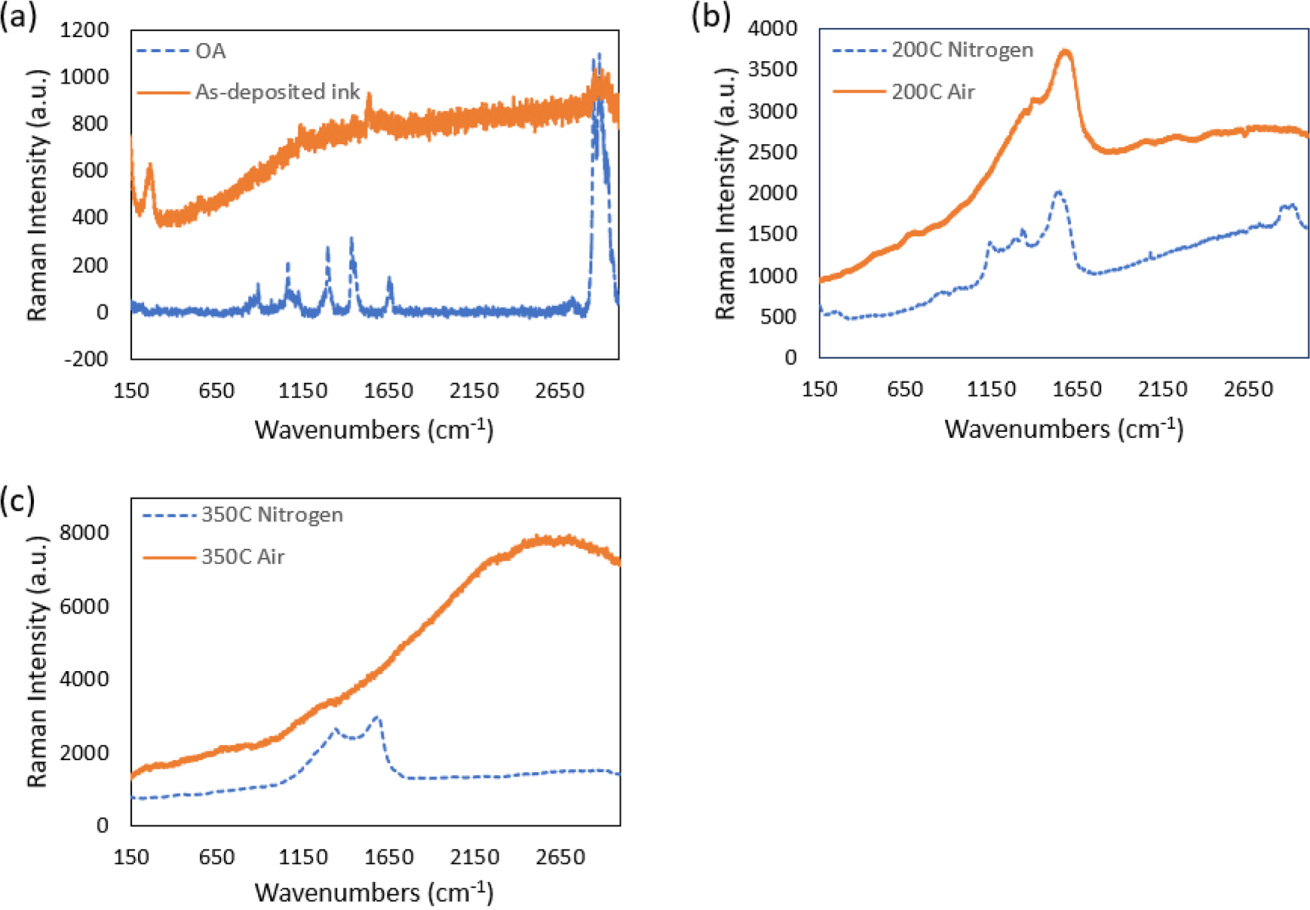
Raman spectra from (a) OA (only), as-deposited ink, and (b) annealed film at 200 °C and in air and N_2_. (c) Annealed film at 200 °C and in air and N_2_. D and G bands of carbon from N_2_-annealed films indicate the presence of nanocrystalline graphitic carbon. Broader peaks and fluorescent background for air-annealed films hiding the D and G bands at sintering temperature (350 °C).

To further shed light on this difference in ligand decomposition, Raman spectroscopy with a 633 nm laser source (Figure 6) was introduced to analyze Raman spectra from OA, as-deposited ink, and different temperature (200 and 350 °C) annealed films in air and N_2_. In Figure 6(a), the as-deposited ink shows characteristic OA peaks around 2800–3000 cm^–1^ corresponding to the C–H vibration. The rest of the OA peaks are not strong enough to be clearly visible under Raman. In Figure 6(b), the spectra of the air-annealed films show disappearing OA peaks and gradual appearance of new broader peaks (more obvious in 200 °C than 350 °C) at ∼1340 cm^–1^ (disordered D band) and ∼1590 cm^–1^ (graphitic G -band) which were generated by the ligand decomposition. For the 350 °C air spectra, the peaks are hard to detect due to the strong fluorescent background. Interestingly, the 200 and 350 °C annealed films in N_2_ in Figure 6(c) show narrower peaks as compared to annealed films in air. The position and intensity ratio of the D band and G band peaks can correlate with the type of residual carbonaceous materials.^56^ The residual carbon can be in different forms, varying from amorphous polymeric hydrogenated carbon (a-C:H) to nanocrystalline graphitic carbon.

For the N_2_ spectra at 200 °C, the first peak is a sharp one at 1332 cm^–1^ with the typical shouldered amorphous carbon peak as the background, and the other one has four separate peaks located at around 1150, 1350, 1500, and 1580 cm^–1^. The peak at around 1150 cm^–1^ is assigned to the nanocrystalline phase of diamond, 1500 cm^–1^ to disordered sp^3^ carbon, and 1350 and 1580 cm^–1^ to the D and G bands.^56^ The 350 °C spectra in N_2_ exhibits strong peaks of D and G bands at around 1330 and 1580 cm^–1^. The position of these two peaks and the I_d_/I_g_ ratio of 0.8957 suggests the transformed OA could be nanocrystalline graphite.^56,57,48^ Recalling Figure 5(b) where OA is completely decomposed at 350 °C (no carbonaceous residue observed by color) in N_2_, it is understood that catalysis of GNP modifies the decomposition of OA in N_2_ by producing residual nanocrystalline graphitic carbon.

For the air-annealed samples Raman spectra, the stronger fluorescent background and the possible broadening of the D and G bands (background is strong enough to hide the true Raman features for the 350 °C film) indicate that the air-annealed spectra (200 and 350 °C) gradually become similar to nanocrystalline graphitic carbon, but it is hard to comment on the exact form of graphitized carbon due to the strong fluorescent background. Besides, the D band intensity around 1350 cm^–1^ for the 200 °C air-annealed film is less (very weak) compared to the N_2_-annealed films (200 and 350 °C). It can be assumed that the disordering of carbon gradually becomes less for air-annealed films with increasing temperature compared to the N_2_ environment. Since the 350 °C air-annealed film shows good electrical conductivity (presented later in this paper), it has higher possibility of containing (short-range ordered) graphitic carbon than amorphous carbon as residue. From the literature, the Raman spectra of the heated OA (in air) at 300 °C^55^ suggests generation of D and G bands (with probable difference in I_d_/I_g_ ratio) which vouch for similar (possibly hidden) peaks in Figure 6(c). It is noted that the onset of the decomposition temperature can be modified using GNP as a catalyst for both environments. The nanocrystalline graphite films are typically produced by annealing at very high temperature (∼900 °C in Ar) after prolonged mechanical scrubbing, deposition at a high temperature, or high-energy post irradiation.^57−59^ Apparently, GNP can control the OA graphitization based on a heating environment and can be used to produce nanocrystalline graphitic carbon with controlled metal content (controlled by OA ratio during GNP synthesis) at low temperature. This guides a new direction for research related to ligand pyrolysis during inorganic nanoparticle synthesis.^55^

**Figure 7:**
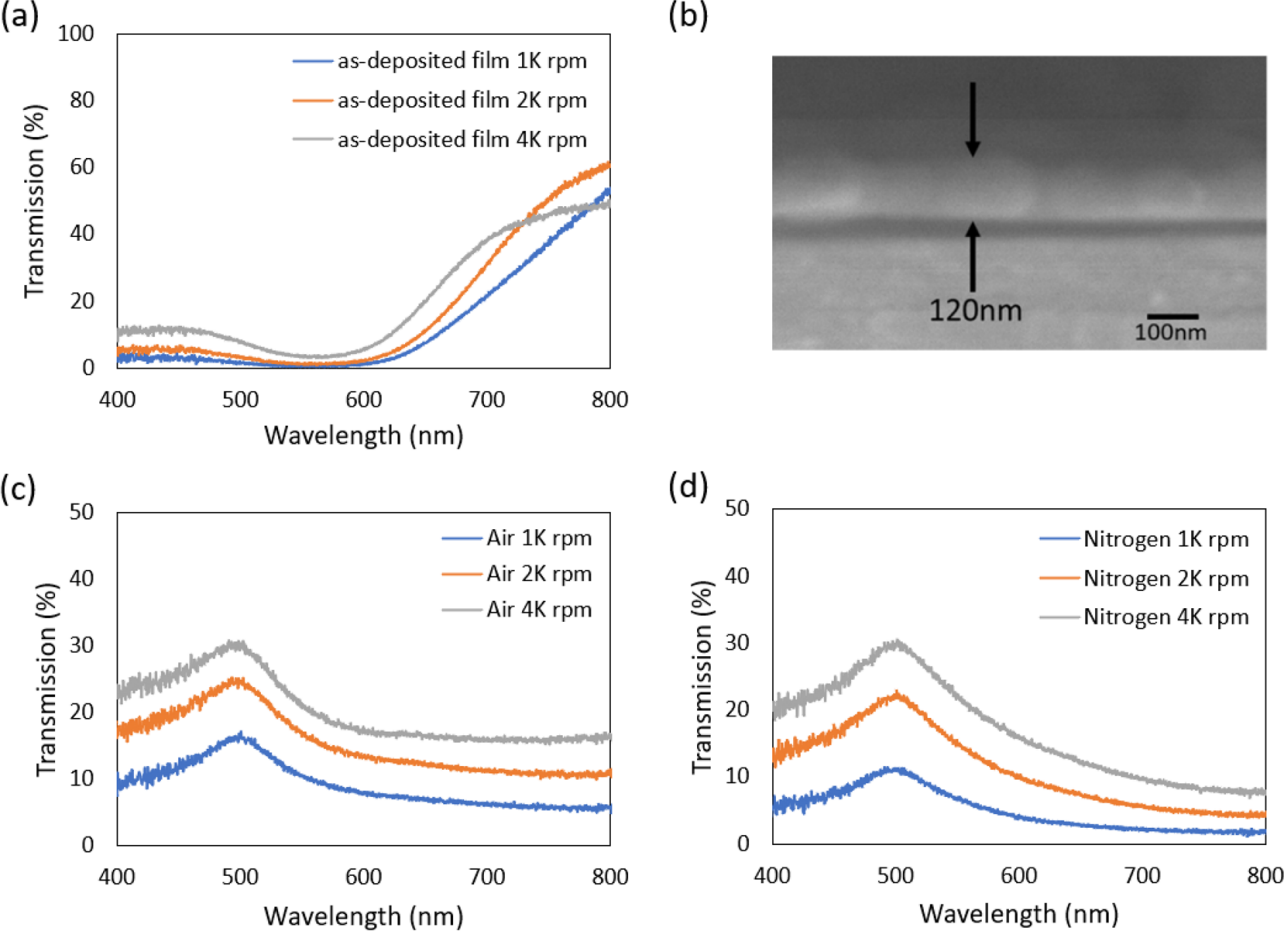
(a) Optical transmission spectra of three different as-deposited film thicknesses (obtained by changing the spin-coating speed) showing reduction in transmission with increasing deposition thickness. (b) Electron micrograph of a representative 120 nm (air-annealed) film. Optical transmission spectra at different thicknesses of the 350 °C annealed film in (c) air and (d) N_2_ environment confirming higher transmission% in air-annealed films than N_2_.

Next, the effect of different annealing environments on the film performance has been tested. Figure 7 depicts the optical transmission spectra of the deposited and annealed films. Three different films were achieved by varying spin-coating speeds (1000, 2000, and 4000 rpm). Optical transmission spectra of the different as-deposited films (before annealing) are depicted in Figure 7(a). Understandably, there is a substantial difference in the optical properties of the resulting gold films. It clearly shows a reduction in transmission with increasing deposition thickness. A localized surface plasmon resonance absorption peak can be identified at 500–600 nm.^60^ The as-deposited films were thermally sintered at 350 °C in both air and N_2_ environments. The obtained thicknesses of the annealed films (in air) are ∼120, ∼103, and ∼92 nm for 1000, 2000, and 4000 rpm spin-coated films, respectively. From the spectra of the annealed films in Figure 7(c and d), it is obvious that the annealed 1000 rpm film exhibits lower transmission than 2000 and 4000 rpm films. Interestingly, for each thickness of the film, air-annealed films are always exhibiting slightly higher transmission compared to N_2_-annealed films indicating that the air-annealed films can be more porous than N_2_-annealed films (as confirmed by SEM later in this paper). A recent study reported that platinum catalysts can induce an oxidation reduction reaction via ligand carbonization, and the opening of the porosity in carbon shell through graphitization is achieved at higher temperature thermal annealing (500–700 °C in N_2_).^61^ In our study, although it is not very clear how the annealing environment can impact the difference in porosity for annealed films, it is suspected that it might be related to the difference in OA graphitization. A detailed investigation will be done in the future to find the reason behind this difference in porosity.

**Figure 8:**
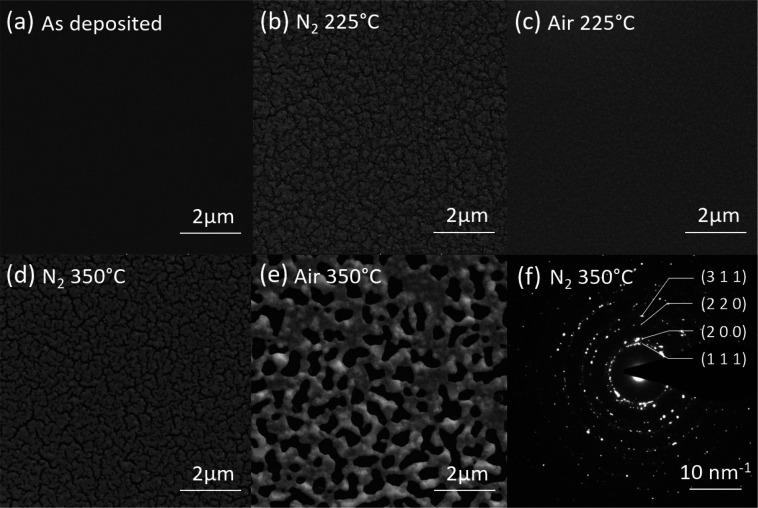
SEM image of (a) as-deposited film, (b) 225 °C N_2_-annealed film, (c) 225 °C air-annealed film, (d) 350 °C N_2_-annealed film, (e) 350 °C air-annealed film, and SAED image of (f) 350 °C N_2_-annealed film.

To observe the morphology and porosity difference of the ink annealing between the environments, three different samples were made. All three samples were spin-coated with GNP ink with 2000 rpm speed, and two of them were anneal at 350 °C in N_2_ and air for 10 min with the aforementioned process. Then, they were investigated with SEM. Figure 8 shows their morphology, and (a) is the as-deposited film, (b) is the N_2_-annealed film, and (c) is the air-annealed film. From Figure 8(a), the as-deposited ink film shows a smooth surface. In Figure 8(b), the film annealed in N_2_ at 225 °C shows that the film is solidified, and there are many small cracks after annealing. This results from the ligand removal and film volume change. In Figure 8(c), the film annealed in air at 225 °C shows only some rough surfaces without any cracks and holes, indicating a relatively small volume change. In Figure 8(d), the film annealed in N_2_ at 350 °C shows wider pores which means higher level ligand decomposition as compared with 225 °C. While in Figure 8(e), the film annealed in air at 350 °C shows larger pores than the N_2_-annealed film. The surface morphology difference also matches the TGA/MS results as presented previously that the ligand decomposition and mass loss are more significant in N_2_ than in air at lower temperatures (<300 °C), while at higher temperatures (>300 °C), the opposite was observed. The formation of pores in the air-annealed sample with a broad distribution of pore sizes ranging from a few hundred nanometers to 1–2 μm contributes to the higher optical transmission in air-annealed samples than that in N_2_-annealed samples at 350 °C.

To determine its gold crystal structure, TEM is performed. Figure 8(f) is the selected area electron diffraction (SAED) image of the 350 °C N_2_-annealed film. The Debye–Scherrer rings indicate a polycrystalline structure of the N_2_-annealed gold film. The adjacent (1 1 1) peaks, (2 0 0) peaks, (2 2 0) peaks, and (3 1 1) peaks indicate the gold is still in FCC structure when heated in a nitrogen environment.^62^

**Figure 9:**
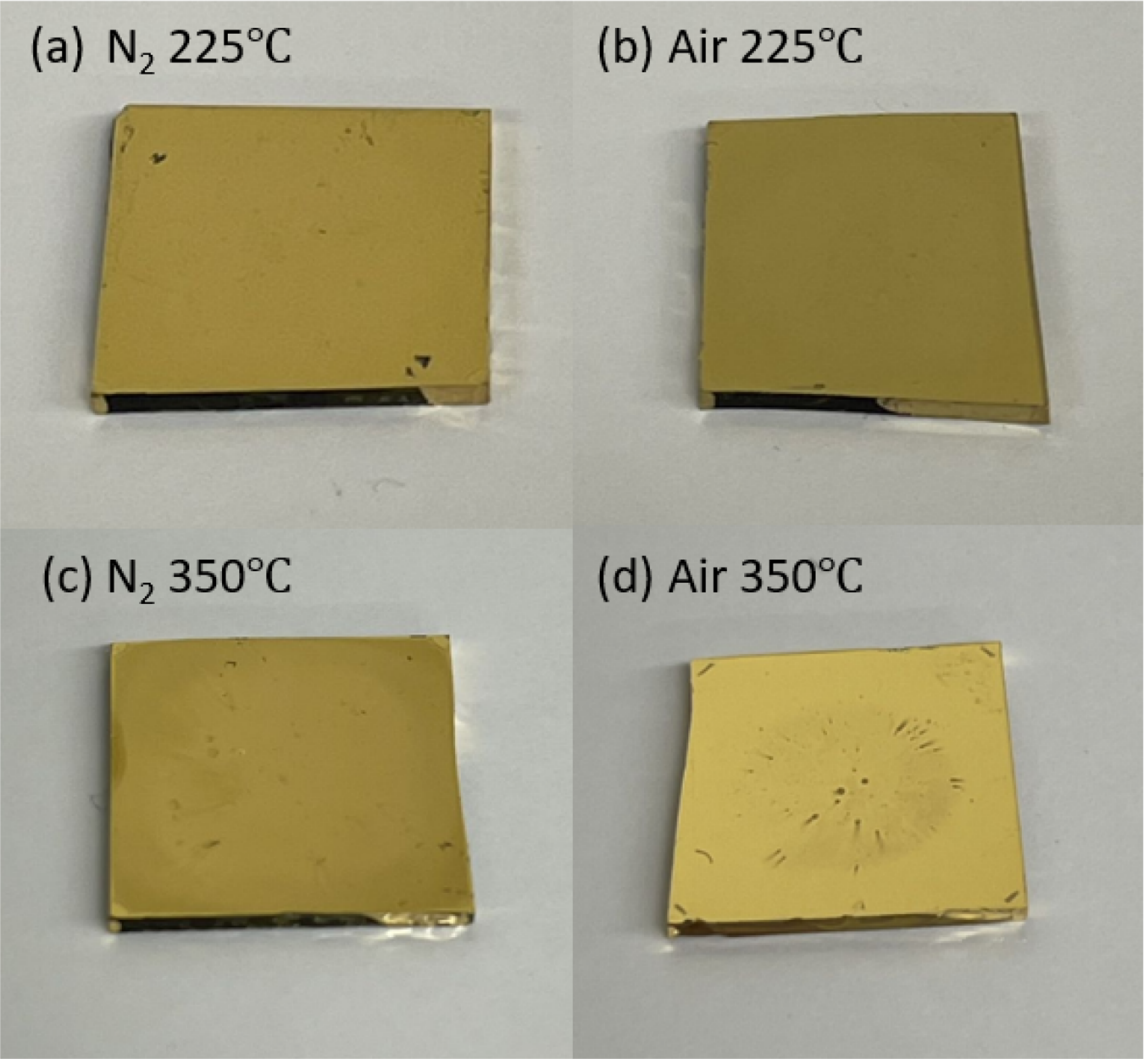
Appearance of (a) 225 °C N_2_-annealed film, (b) 225 °C air-annealed film, (c) 350 °C N_2_-annealed film, and (d) 350 °C air-annealed film.

The appearance of air-annealed and N_2_-annealed OA-GNP films can be seen in Figure 9. OA-GNP films show a similar dark golden color when annealed at 225 °C in N_2_ and in air (Figure 9(a and b)), as well as annealed at 350 °C in N_2_ (Figure 9(c)), which is in accordance with the low porosity samples as indicated by SEM images in Figure 8(b–d). In contrast, the film annealed in air at 350 °C (Figure 9(d)) shows a brighter golden color, which is in accordance with the pore formation in the annealed film shown in Figure 8(e).

**Figure 10:**
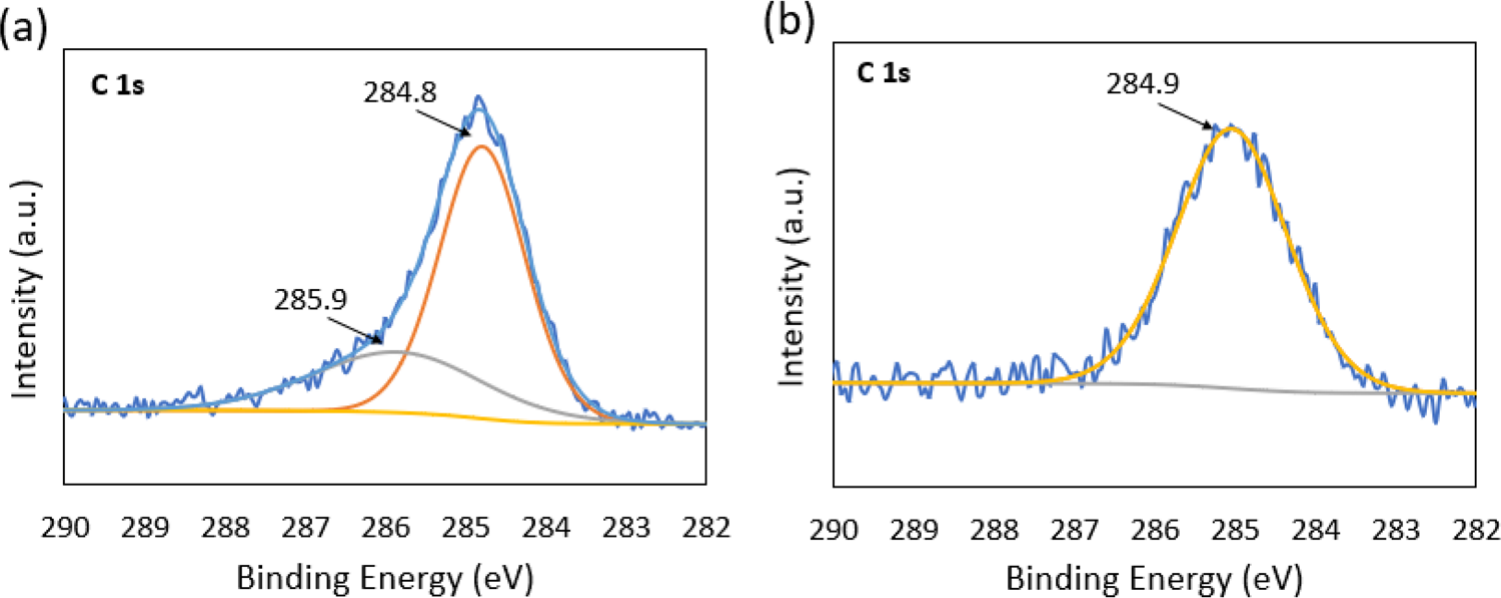
XPS of C 1s at (a) N_2_-annealed film and (b) air-annealed film.

To understand the difference of graphite on the film surfaces between N_2_- and air-annealed films, XPS was used to find details of carbon binding. Figure 10 shows the XPS of C 1s of (a) N_2_-annealed GNP ink and (b) air-annealed GNP ink. There are two peaks located at 284.8 and 285.9 eV in the N_2_-annealed film, which represents sp^2^ and sp^3^ carbon hybridization on the surface. The appearance of sp^3^ carbon hybridization indicates the existence of disordered graphite, while in Figure 10(b) there is only one peak at 284.9 eV in the air-annealed film which represents sp^2^ carbon hybridization indicating the presence of ordered graphitic carbon in the air-annealed sample.^63^ The XPS comparison revealed that the air-annealed film generates much more ordered graphitic carbon than the N_2_-annealed film.

In general, a graphitic structure has higher density than that of amorphous carbonaceous matrix.^64^ Obtaining porous carbon via catalytic graphitization has recently been attempted.^65^ Besides, the transformation of amorphous carbon to graphitic carbon without any catalyst addition is typically very difficult to achieve and involves very high temperature (beyond 2000 °C).^64^ In our study, the observed difference in graphitization from Raman at both air and N_2_ environments should be investigated more in the future to harness any possibility of controlled catalytic graphitization at low temperature.

**Figure 11:**
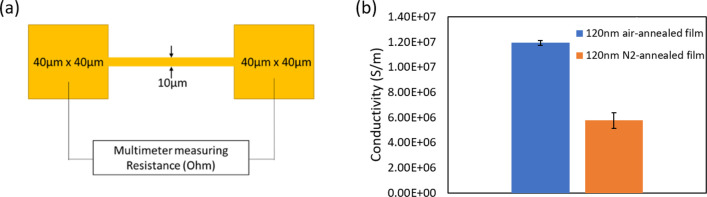
(a) Schematic of the laser-machined pads and resistance measuring setup. (b) Normalized conductivity values from the setup for two films in different environments.

To understand how the OA graphitization and film porosity direct the electrical conductivity of the sintered films, a two-point probe setup was used to generate data for measured resistances (between the two probes) on 350 °C air-annealed films with 120 nm thickness in two different environments (air and N_2_). Figure 10 depicts the gathered data from the two-point probe test. Laser machining was used to machine pads with a fixed dimension (40 μm × 40 μm pads either 50 or 100 μm apart with a 10 μm wide line connecting the two pads) on the as-deposited film, followed by 350 °C annealing for 2 min in air and N_2_, respectively. Figure 11(a) depicts the schematic of the two-point probe measurement setup, where the two probes (connected to multimeter) contact the premachined (and annealed) pads to give reading in ohm. It was observed that films annealed below 200 °C show MΩ resistances for both thickness and heating environment. In between 200 and 250 °C, the resistances start to drop, and finally, at 350 °C, the lowest resistances are recorded for all the films. Hence, 350 °C annealing was used to measure the resistance for the films with different thickness. Since the two-point probe setup has some unavoidable background (and contact resistance), two different lengths (100 and 50 μm) of the machined lines were used to determine the difference in resistance from it and calculate the film conductivity. It was determined that the air-annealed film exhibited conductivity of ∼1.2 × 10^7^ S/m, which is ∼29% of bulk gold, and comparatively, the N_2_-annealed films exhibited lower conductivity with ∼5.8 × 10^6^ S/m which is ∼14% of bulk gold. The result is shown in Figure 11(b). Interestingly, for the N_2_-annealed films, the measured resistance from the same setup is ∼1 time lower than that of the air-annealed one. This hints that the difference in OA graphitization (in air and N_2_ environments) and film porosity can affect the GNP sintering and final film conductivity. Generally, amorphous carbon is not conductive, and the higher the degree of graphitization (ordered) is, the higher the electrical conductivity is. Since the air-annealed films show good electrical conductivity, it can be safely assumed that the residual carbon could be more (ordered) graphitic than amorphous. Reports are found in the literature that mention achieving ordered graphitization via metal catalysis.^66,67^ It is postulated that for the air-annealed films the residual carbon could be short-range ordered graphitic (at low temperature ∼350 °C, higher or complete ordering might not be possible), making it possible to attain better conductivity compared with N_2_-annealed films despite higher porosity.

**Scheme 1:**
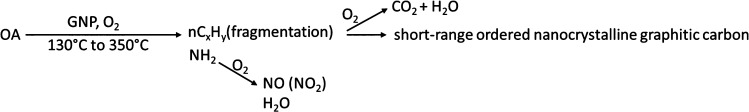
Proposed Reaction Scheme for Air Annealing of OA-Capped GNP

Finally, the understanding until now needs to be summed up to propose possible reaction schemes of OA-capped GNP at air and N_2_. A description of thermal decomposition products for OA in air could not be found, but for a similar molecule, CTAB (cetyltrimethylammonium bromide), the ammonium headgroup is first removed by the Hofmann degradation process first. Then, the cracking of the remaining hydrocarbon chain is attained up to 600 °C.^65^ The TGA curve of this literature resembles the TGA of OA in air and thus indicates the possible air decomposition products of OA. Hence, the proposed decomposition scheme for air annealing of the OA-capped GNP is described in Scheme 1.

Scheme 1 proposes that above 130 °C (and up to 350 °C) in air, catalyzed decomposition of OA occurs through initial oxidation of an amine group (converts into NO, NO_2_, and H_2_O) and fragmentation of the OA chain. The fragmented OA chain can further oxidize into CO_2_ and H_2_O, additionally converting into a short-range ordered nanocrystalline graphitic carbon residue (ordering and graphitization of amorphous carbon is enhanced in the presence of oxidizing gases^68^).

**Scheme 2:**
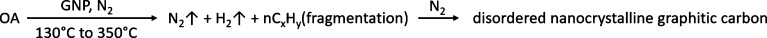
Proposed Reaction Scheme for N_2_ Annealing of OA-Capped GNP

The proposed decomposition scheme for the N_2_ annealing of OA-capped GNP is described in Scheme 2.

Scheme 2 proposes that above 130 °C (and up to 350 °C) in N_2_, catalyzed decomposition of OA occurs through initial fragmentation of the OA chain and subsequent conversion into higher resistance nanocrystalline graphitic carbon (comparatively less ordered than Scheme 1). A catalytic reduction reaction might induce formation of N_2_ from the amine group of OA. The H_2_ generated from the amine group can provide potential reduction reaction sites.^51^ Correlating the TGA curve from Figure 3 with the proposed Scheme 2, it can be postulated that due to GNP catalysis the hydrocarbon chain is fragmented, generating N_2_ and H_2_ which could potentially contribute to more mass loss in N_2_ compared to air annealing.

## Conclusions

In this paper, we investigated thermal sintering of OA-capped GNP in air and N_2_ environments. FTIR confirmed the presence of OA as a capping ligand in the ink. TGA revealed a distinct mass loss difference for both environments. The GNP can act as a catalyst which reduces the OA decomposition onset temperature by ∼50 °C in N_2_ and ∼110 °C in air compared to OA. Raman showed the differences in D and G band appearance indicating nanocrystalline graphitic carbon for air and N_2_ annealing. Later, optical transmission spectroscopy and two-point probe measurements identified the air annealing to have higher porosity with lower resistance compared to N_2_. All these differences were understood to be evolving due to the catalytic ordered graphitization at low temperature. Finally, two separate reaction schemes have been proposed for the two annealing environments. This work provides insights into potential pathways for obtaining size-controlled nanocrystalline graphitic carbon at lower temperature though catalytic decomposition of ligands in specified environments.
